# Association of *ApaI* rs7975232 and *BsmI* rs1544410 in clinical outcomes of COVID-19 patients according to different SARS-CoV-2 variants

**DOI:** 10.1038/s41598-023-30859-7

**Published:** 2023-03-03

**Authors:** Ayad Naji Radha Al-Gharrawi, Enayat Anvari, Abolfazl Fateh

**Affiliations:** 1 Department of Biology, Science and Research Branch, Islamic Azad University, Tehran, Iran; 2 Clinical Research Development Unit, Shahid Mostafa Khomeini Hospital, Ilam University of Medical Science, Ilam, Iran; 3 Department of Mycobacteriology and Pulmonary Research, Pasteur Institute of Iran, Tehran, Iran; 4 Microbiology Research Center (MRC), Pasteur Institute of Iran, Tehran, Iran

**Keywords:** SARS-CoV-2, SARS virus

## Abstract

A growing body of research has shown how important vitamin D is in the prognosis of coronavirus disease 19 (COVID-19). The vitamin D receptor is necessary for vitamin D to perform its effects, and its polymorphisms can help in this regard. Therefore, we aimed to evaluate whether the association of *ApaI* rs7975232 and *BsmI* rs1544410 polymorphisms in different severe acute respiratory syndrome coronavirus 2 (SARS-CoV-2) variants were influential in the outcomes of COVID-19. The polymerase chain reaction-restriction fragment length polymorphism method was utilized to determine the different genotypes of *ApaI* rs7975232 and *BsmI* rs1544410 in 1734 and 1450 patients who had recovered and deceased, respectively. Our finding revealed that the *ApaI* rs7975232 AA genotype in the Delta and Omicron BA.5 and the CA genotype in the Delta and Alpha variants were associated with higher mortality rate. Also, the *BsmI* rs1544410 GG genotype in the Delta and Omicron BA.5 and the GA genotype in the Delta and Alpha variants were related to a higher mortality rate. The A-G haplotype was linked with COVID-19 mortality in both the Alpha and Delta variants. The A-A haplotype for the Omicron BA.5 variants was statistically significant. In conclusion, our research revealed a connection between SARS-CoV-2 variants and the impacts of *ApaI* rs7975232 and *BsmI* rs1544410 polymorphisms. However, more research is still needed to substantiate our findings.

## Introduction

The World Health Organization (WHO) reports demonstrate that severe acute respiratory syndrome associated with coronavirus-2 (SARS-CoV-2) has caused Coronavirus disease 2019 (COVID-19), a worldwide pandemic, in millions of people since December 2019. The symptoms of the COVID-19 can range from mild to moderate and severe to critical, necessitating hospitalization and intensive care unit (ICU) admissions for some patients^1^. It is appropriate to shift our focus from preventive to therapeutic measures in light of the development of effective vaccines against COVID-19, whose efficacies are unusually high but not absolute, and in light of the prospect that new viral variations may limit its efficacy. Additionally, there are still a significant number of unvaccinated people^2^.

Vitamin D deficiency has increased the severity of viral illnesses such as influenza^3^. According to recent research, vitamin D can influence SARS-CoV-2 gene expression and reduce infection rate when it binds to the vitamin D response element^4^. The angiotensin-converting enzyme 2 (ACE2) mediates the SARS-CoV-2 infection and its receptor, which vitamin D. Additionally regulates, vitamin D is known to increase the generation of antimicrobial proteins, modulate innate and adaptive immune responses, and possibly operate as an anti-inflammatory agent^5–7^.

Vitamin D exerprimarily biological effects through vitamin D receptors (VDRs) are mostly found in the gastrointestinal tract, bones, lungs, and most immune cells. Even though the VDR is abundantly expressed in the lung tissue, it is yet unclear how vitamin D-VDR signaling may contribute to pulmonary immunopathology^8^.

Regarding the related mechanisms, single nucleotide polymorphisms (SNPs) in the gene encoding the *VDR* could alter the protective efficacy of vitamin D-mediated host responses. This may be done, for instance, by affecting the structure of the *VDR*, which will impact the transcription of genes regulated by vitamin D that affect immune function^9^. *VDR* polymorphisms have been linked to an increased risk of acute lower respiratory infections in various contexts^10–12^. Several studies have investigated the association between four *VDR* polymorphisms including, *TaqI* (rs731236; exon 9; A > G), *Fok*I (rs2228570; exon 2; C > T), *Apa*I (rs7975232; intron 8; C > A), and *Bsm*I (rs1544410; intron 8; G > A) and the risk of hepatitis B virus infection in different ethnic groups^13,14^.

It is important to note that the results of genetic studies investigating the function of the *ApaI* rs7975232 and *BsmI* rs1544410 polymorphisms in the pathogeneses of COVID-19 remained controversial. Therefore, this study aimed to examine whether these *ApaI* rs7975232 and *BsmI* rs1544410 polymorphisms play a role in the susceptibility to the COVID-19 of different variants of SARS-CoV-2.

## Materials and methods

### Sample collection

We confirm that all experimental protocols were approved by an Ilam University of Medical Science ethical committee. Moreover, all methods were performed in accordance with the relevant guidelines and regulations.

From 14,117 patients who visited a hospital of Ilam University of Medical Sciences between November 2020 to February 2022 during the three peaks (Alpha, Delta, and Omicron BA.5) of the SARS-CoV-2 infection, 3184 patients were selected based on the following criteria: (1) having a positive real-time reverse transcription polymerase chain reaction (rtReal time-PCR) from the pharyngeal swab samples that were selected from a hospital; (2) giving informed consent to participate in the study; (3) having Iranian nationality with the same ethnicity; (4) lack of underlying comorbidities including pulmonary infection (cystic fibrosis, chronic obstructive pulmonary disease, and asthma), liver disease, chronic kidney disease, heart disease (cardiovascular disease, heart failure, and etc.), cancer, immunocompromised disease (transplant patients and human immunodeficiency virus), hypertension, pregnancy, and diabetes.

In this study, we examined two groups. One was patients with mild and moderate symptoms (cough, malaise, loss of taste and smell, fever, muscle pain, sore throat, nausea, diarrhea, vomiting, headache, and oxygen saturation (SpO_2_) above 94% on room air at sea level), which were considered as the control group (recovered patients), and the second group was patients with severe and critical symptoms (SpO_2_ of 94% below room air at sea level, PaO_2_/FiO_2_ of 300 mm Hg, lung infiltrates less than 50%, septic shock, difficulty breathing during slight movement or even at rest and multiple organ dysfunction) as the case group (deceased patients).

All paraclinical information such as lipid profile, liver enzymes, complete blood count (CBC), real-time PCR cycle threshold (Ct) values, 25-hydroxyvitamin D, C-reactive protein (CRP), uric acid, erythrocyte sedimentation rate (ESR), and creatinine were obtained when visiting the hospital.

### *ApaI* rs7975232 and *BsmI* rs1544410 genotyping

After DNA extraction of all patients using the High-pure PCR Template Preparation Kit (Roche Diagnostics Deutschland GmbH, Mannheim, Germany), *ApaI* rs7975232 and *BsmI* rs1544410 genotyping was performed using polymerase chain reaction-restriction fragment length polymorphism (PCR–RFLP) method.

The forward and reverse sequence primers for *ApaI* rs7975232 with the PCR product sizes 242 bp included 5'-CTGCCGTTGAGTGTCTGTGT-3' and 5'-TCGGCTAGCTTCTGGATCAT-3', respectively. The forward and reverse sequence primers for *BsmI* rs1544410 with the PCR product sizes 297 bp were 5'-GGGAGACGTAGCAAAAGGAG-3' and 5'-CCATCTCTCAGGCTCCAAAG-3', respectively. The PCR conditions were following: initial denaturation at 95 °C for 5 min, followed by 35 cycles of 95 °C for 30 s, 57 °C for 30 s, 72 °C for 35 s, and final extension at 72 °C for 10 min was for *ApaI* rs7975232 and initial denaturation at 95 °C for 5 min, followed by 35 cycles of 95 °C for 30 s, 58 °C for 30 s, 72 °C for 45 s, and final extension at 72 °C for 10 min was for *BsmI* rs1544410.

The PCR products were digested with *ApaI* and *BsmI*, according to the manufacturer's instructions, and were visualized by electrophoresis on 2.5% agarose gel. The product sizes for *ApaI* rs7975232 after digestion were 191 bp and 51 bp for the CC genotype and 242 bp for the AA genotype, and for *BsmI* rs1544410, were 192 bp and 105 bp for the GG genotype and 297 bp for the AA genotype^15^.

For the PCR–RFLP result confirmation, several samples were randomly selected and sequenced on an ABI 3500 DX Genetic Analyzer (ABI, Thermo Fisher Scientific, Waltham, MA, USA) by the Sanger sequencing method. Then raw data were analyzed with ChromasPro software.

### Statistical analyses

SPSS version 22.0 (SPSS, Inc, Chicago, IL, USA) was used for analysis. The Chi-square test was used to evaluate the significance of the relationship between the two qualitative groups. The Shapiro–Wilk test was used to determine the distribution's normality, and Mann–Whitney U test was used for quantitative data.

The Chi-square test was used to examine all SNPs for Hardy–Weinberg equilibrium (HWE). Using SNPStats software, the correlation analysis was carried out, including dominant, over-dominant, co-dominant and recessive models. The minor allele frequency (MAF) and linkage disequilibrium (LD) was also determined. The fitting-best model was chosen using the Akaike Information Criterion (AIC) and the Bayesian Information Criterion (BIC). The most effective model was the one with the lowest AIC score (http://bioinfo.iconcologia.net/SNPStats). Logistic regression was used to determine odds ratios (ORs) and their respective 95% confidence intervals (CIs) for each model. *P*-values lower than 0.05 were deemed significant.

## Results

### Baseline clinical features and demographics

Table 1 demonstrates the characteristics of the study participants. Three variants of SARS-CoV-2 were included in the study. Among 3184 patients, there were 1022 Alpha variant, 1026 Delta variant, and 1132 Omicron BA.5 variant. The mean age of the patients with the Delta variant (58.0 ± 11.8) was higher than those with Alpha (53.0 ± 12.7) and Omicron BA.5 (53.7 ± 12.9) variants. The number of males and females in the Alpha variant was 479 (46.9%) and 543 (53.1%), respectively. In the Delta variant, these numbers were 546 (53.2%) and 480 (46.8%) and in the Omicron BA.5 variant, they were 546 (53.2%) and 480 (46.8%), respectively.

**Table 1:** Comparison of laboratory parameters between SARS-CoV-2 variants.

Variables	SARS-CoV-2 variants			*P*-value
	Alpha (n = 1022)	Delta (n = 1026)	Omicron BA.5 (n = 1136)	
Deceased/ Improved patients	479/543 (46.9/53.1%)	674/352 (65.7/34.3%)	297/839 (26.1/73.9%)	< 0.001*
Mean age ± SD	53.0 ± 12.7	58.0 ± 11.8	53.7 ± 12.9	0.128
Gender (male/female)	525/497 (51.4/48.6%)	546/480 (53.2/46.8%)	598/538 (52.6/47.4%)	0.692
ALT, IU/L (mean ± SD) (Reference range: up to 41)	38.5 ± 24.8	40.8 ± 24.7	35.8 ± 24.2	0.001
AST, IU/L (mean ± SD) (Reference range: up to 38)	34.9 ± 15.5	34.5 ± 14.0	31.9 ± 14.4	< 0.001*
ALP, IU/L (mean ± SD) (Reference range: 90–450)	190.2 ± 84.7	188.6 ± 74.0	177.2 ± 83.5	< 0.001*
Cholesterol, mg/dL (mean ± SD) (Reference range: up to 200)	116.1 ± 34.1	120.5 ± 40.5	123.1 ± 39.4	< 0.001*
TG, mg/dL (mean ± SD) (Reference range: up to 200)	124.1 ± 54.9	121.6 ± 48.8	126.9 ± 55.9	0.245
LDL, mg/dL (mean ± SD) (Reference range: up to 150)	82.8 ± 45.1	85.3 ± 45.3	104.7 ± 48.3	< 0.001*
HDL, mg/dL (mean ± SD) (Reference range: 35–70)	32.5 ± 11.3	32.1 ± 11.5	33.6 ± 11.7	0.039*
WBC, 10^9^/L (mean ± SD) (Reference range: 4000–10,000)	7627.3 ± 2843.2	7599.2 ± 2715.7	7704.9 ± 2807.7	0.297
CRP, mg/L (mean ± SD) (Reference range: < 10 mg/L Negative)	61.6 ± 21.5	63.9 ± 22.0	60.2 ± 21.7	0.122
ESR, mm/1st h (mean ± SD) (Reference range: 0–15)	50.1 ± 16.0	52.3 ± 16.0	49.1 ± 16.1	0.025
FBS, mg/dL (mean ± SD) (Reference range: 70–100)	107.1 ± 41.6	109.8 ± 43.2	106.5 ± 40.7	0.716
Platelets × 1000/cumm (mean ± SD) (Reference range: 140,000–400,000)	184 ± 71	185 ± 74	184 ± 69	0.994
Uric acid, mg/dL (mean ± SD) (Reference range: 3.6–6.8)	4.8 ± 1.8	4.4 ± 1.7	5.2 ± 1.8	< 0.001*
Creatinine, mg/dL (mean ± SD) (Reference range: 0.6–1.4)	0.9 ± 0.3	1.0 ± 0.3	0.8 ± 0.3	< 0.001*
qPCR Ct value	20.1 ± 6.4	17.4 ± 6.1	21.9 ± 6.0	< 0.001*
25-hydroxy vitamin D, ng/mL (mean ± SD) (Sufficiency: > 30)	24.2 ± 12.8	21.8 ± 10.3	33.0 ± 13.4	0.029*

*ALT* alanine aminotransferase, *AST* aspartate aminotransferase, *ALP* alkaline phosphatase, *TG* triglyceride, *LDL* low density lipoprotein, *HDL* high density lipoprotein, *WBC* white blood cells, *CRP* C-reactive protein, *ESR* erythrocyte sedimentation rate, *FBS* fasting blood glucose, *SD* standard deviation, *SARS-CoV-2* Severe Acute Respiratory Syndrome Coronavirus 2.

*Statistically significant (< 0.05).

The 25-hydroxy vitamin D rates in the Alpha, Delta, and Omicron BA.5 variants were (24.2 ± 12.8), (21.8 ± 10.3), and (33.0 ± 13.4), respectively, which was significant between variants (*P* = 0.029). The mean qPCR Ct values in the Delta variant (17.4 ± 6.1) were higher than the Alpha (20.1 ± 6.4) and Omicron BA.5 (21.9 ± 6.0) variants (*P* < 0.001).

### Relationship between COVID-19 mortality adjusted by SARS-CoV-2 variants and *ApaI* rs7975232 and *BsmI* rs1544410 polymorphisms

The COVID-19 death rate was considerably more significant in patients with the *ApaI* rs7975232 AA genotype than in other genotypes. Patients who have recovered from COVID-19 also had the *ApaI* rs7975232 CC genotype. Patients with the GG genotype exhibited a higher COVID-19 mortality rate in the *BsmI* rs1544410 polymorphism.

Table 2 tabulates the inheritance model analysis results for *ApaI* rs7975232 and *BsmI* rs1544410 polymorphisms in patients. By comparing the deceased and recovered patients, the codominant and dominant inheritance models with the lowest AIC and BIC values were found to be the best-fitting models for *ApaI* rs7975232 and *BsmI* rs1544410. The *ApaI* rs7975232 AA genotype was linked to a higher risk of COVID-19 mortality (*P* < 0.0001, OR 1.87, 95% CI 1.49–2.35), whereas the *BsmI* rs1544410 GA/GG genotype was associated with a higher risk of COVID-19 mortality (*P* < 0.0001, OR 1.97, 95% CI 1.68–2.30).

**Table 2:** *ApaI* rs7975232 and *BsmI* rs1544410 polymorphisms association with COVID-19 mortality adjusted by SARS- CoV-2 variants.

Model	Genotype		Groups					
			Recovered patients	Deceased patients	OR (95% CI)	*P*-value	AIC	BIC
*ApaI* rs7975232								
Allele	C		2346 (68.0%)	1675 (58.0%)	–	–	–	–
	A		1122 (32.0%)	1225 (42.0%)	–	–	–	–
Codominant	C/C		796 (45.9%)	514 (35.5%)	1.00	< 0.0001*	4020.0	4056.4
	C/A		754 (43.5%)	647 (44.6%)	1.16 (0.99–2.35)			
	A/A		184 (10.6%)	289 (19.9%)	1.87 (1.49–2.35)			
Dominant	C/C		796 (45.9%)	514 (35.5%)	1.00	< 0.0001*	4035.7	4066.0
	C/A–A/A		938 (54.1%)	936 (64.5%)	1.73 (1.12–1.52)			
Recessive	C/C–C/A		1550 (89.4%)	1161 (80.1%)	1.00	< 0.0001*	4021.3	4051.6
	A/A		184 (10.6%)	289 (19.9%)	1.73 (1.40–2.13)			
Overdominant	C/C–A/A		980 (56.5%)	803 (55.4%)	1.00	0.770	4047.5	4077.8
	C/A		754 (43.5%)	647 (44.6%)	0.98 (0.84–1.13)			
Minor allele frequency (A)			0.32	0.42	–	–	–	–
*BsmI* rs1544410								
Allele	A		2414 (70.0%)	1770 (61.0%)	–	–	–	–
	G		1054 (30.0%)	1130 (39.0%)	–	–	–	–
Codominant	A/A		797 (46.0%)	479 (33.0%)	1.00	< 0.0001*	3974.0	4010.4
	G/A		820 (47.3%)	812 (56.0%)	1.94 (1. 65–2.22)			
	G/G		117 (6.8%)	159 (11.0%)	2.12 (1.60–2.81)			
Dominant	A/A		797 (46.0%)	479 (33.0%)	1.00	< 0.0001*	3972.4	4002.7
	G/A–G/G		937 (54.0%)	971 (67.0%)	1.97 (1.68–2.30)			
Recessive	A/A–G/A		1617 (93.2%)	1291 (89.0%)	1.00	0.0044*	4039.4	4069.8
	G/G		117 (6.8%)	159 (11.0%)	1.47 (1.13–1.91)			
Overdominant	A/A–G/G		914 (52.7%)	638 (44.0%)	1.00	< 0.0001*	4000.0	4030.4
	G/A		820 (47.3%)	812 (56.0%)	1.69 (1.46–1.97)			
Minor allele frequency (G)			0.30	0.39	–	–	–	–

*COVID-19* coronavirus disease, *SARS-CoV-2* Severe Acute Respiratory Syndrome Coronavirus 2, *OR* odds ratios, *CI* confidence intervals, *AIC* Akaike information criterion, *BIC* Bayesian information criterion, *OR* odds ratios, *CI* confidence intervals.

*Statistically significant (< 0.05).

The *ApaI* rs7975232 polymorphism in recovered and deceased patients was compatible with HWE (*P* > 0.05), while HWE in *BsmI* rs1544410 was incompatible in both groups (*P* < 0.001). The MAF for *ApaI* rs7975232 (A) and *BsmI* rs1544410 (G) polymorphisms in deceased patients was higher than in recovered patients.

### Correlation between the value of vitamin D and *ApaI* rs7975232 and *BsmI* rs1544410 polymorphism

**Figure 1:**
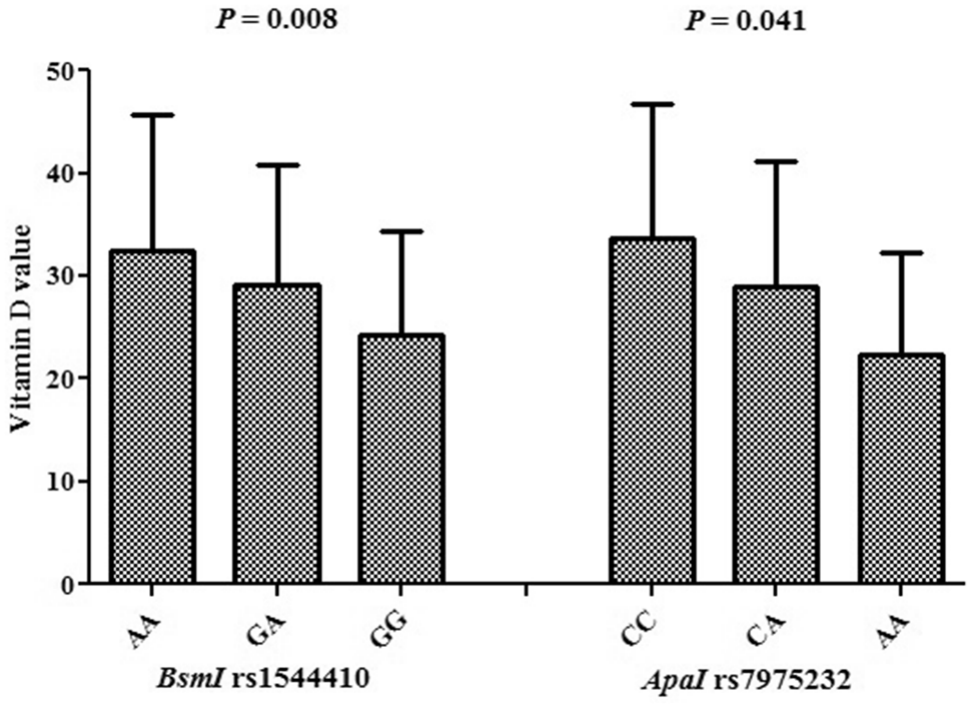
Vitamin D levels as per genotypic distribution of *BsmI* rs1544410 and *ApaI* rs7975232 polymorphisms.

The relation between vitamin D values and different genotype frequencies is indicated in Fig. 1. There is significant difference in vitamin D levels between different *ApaI* rs7975232 (*P* = 0.041) and *BsmI* rs1544410 (*P* = 0.008) genotypes among recovered and deceased patients. The lowest amount of vitamin D was found in *ApaI* rs7975232 GG and *BsmI* rs1544410 AA genotypes, while the highest amount in *ApaI* rs7975232 AA and *BsmI* rs1544410 CC genotypes.

### Frequencies of *ApaI* rs7975232 and *BsmI* rs1544410 polymorphism between SARS-CoV-2 variants

Our findings showed that the death rate was related to the SARS-CoV-2 variants, much higher in the Delta variant than in the Alpha and Omicron BA.5 variants (*P* < 0.001).

**Figure 2:**
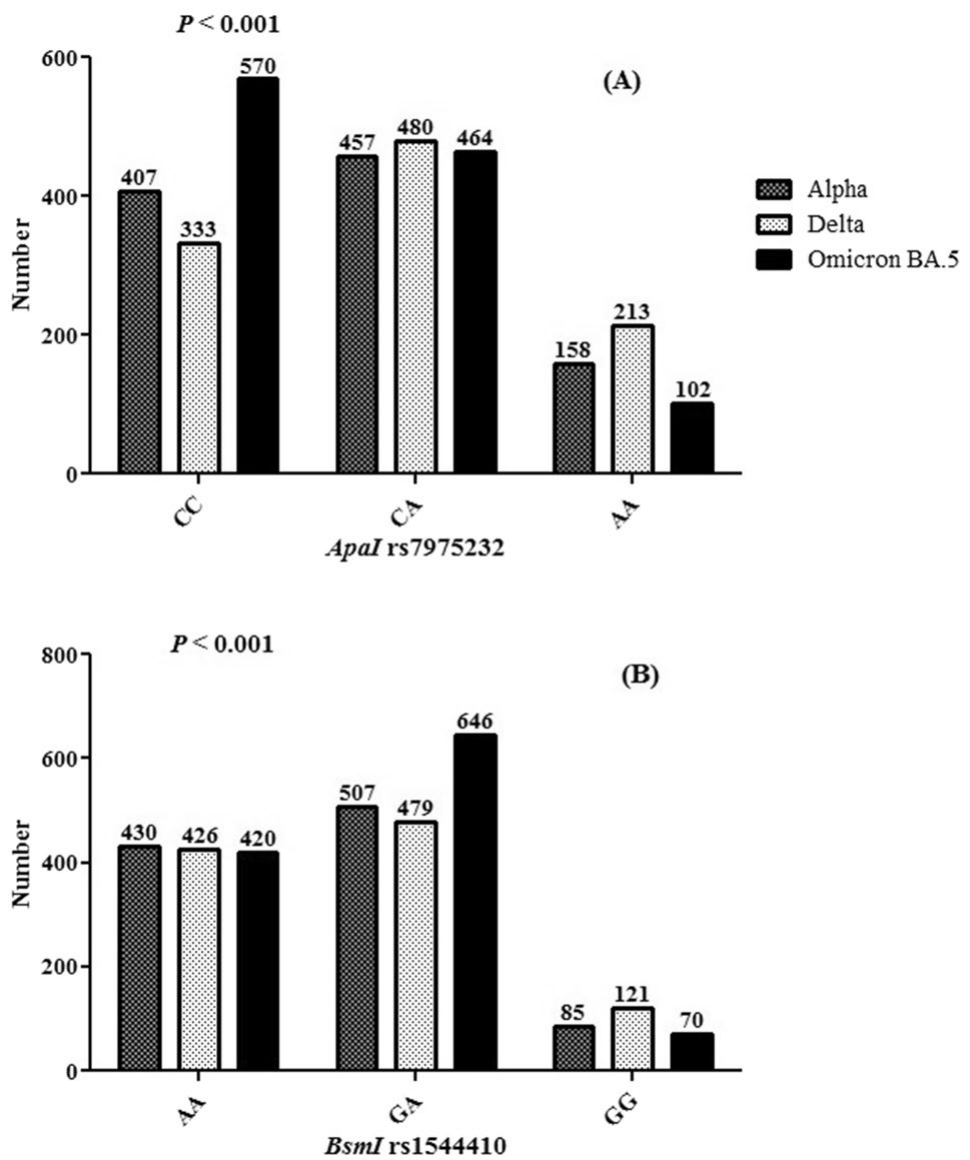
(**A**) The frequency of *ApaI* rs7975232 genotypes and (**B**) *BsmI* rs1544410 genotypes according to SARS-CoV-2 variants.

The frequencies of CC, CA, and AA genotypes in *ApaI* rs7975232 polymorphism in the Alpha variant were 407 (39.8%), 457 (44.7%), and 158 (15.5%), respectively. These frequencies in the Delta variant was 333 (32.5%), 480 (46.8%), and 213 (20.7%), respectively. In the Omicron BA.5 variant, the frequency of CC was 570 (50.2%), CT was 464 (40.8%), and TT was 102 (9.0%) (Fig. 2A).

When we adjusted the effect of *ApaI* rs7975232 polymorphism for SARS-CoV-2 variants, the COVID-19 mortality rate was related to *ApaI* rs7975232 CA (OR 1.32, 95% CI 1.01–1.73) in the Alpha variant and with *ApaI* rs7975232 AA (OR 3.03, 95% CI 2.09–4.41*)* and CA (OR 2.97, 95% CI 2.21–3.99*)* in the Delta variant and with AA in the Omicron BA.5 variant (OR 3.86, 95% CI 2.49–5.99) (Table 3).

**Table 3:** *ApaI* rs7975232 and *BsmI* rs1544410 genotypes association with SARS-CoV-2 variants.

Variants	rs7975232 genotypes	Recovered patients	Deceased patients	OR (95% CI)
Alpha	C/C	228	179	1.00
	C/A	224	233	1.32 (1.01–1.73)
	A/A	91	67	0.94 (0.65–1.36)
Delta	C/C	171	162	1.00
	C/A	126	354	2.97 (2.21–3.99)
	A/A	55	158	3.03 (2.09–4.41)
Omicron BA.5	C/C	397	173	1.00
	C/A	404	60	0.82 (0.73–1.01)
	A/A	38	64	3.86 (2.49–5.99)

Variants	rs1544410 Genotypes	Recovered patients	Deceased patients	OR (95% CI)
Alpha	A/A	291	139	1.00
	G/A	198	309	3.26 (2.49–4.27)
	G/G	54	31	1.20 (0.74–1.95)
Delta	A/A	230	196	1.00
	G/A	90	389	3.26 (2.09–5.10)
	G/G	32	89	5.08 (3.77–6.84)
Omicron BA.5	A/A	276	144	1.00
	G/A	532	114	0.86 (0.59–1.06)
	G/G	31	39	2.41 (1.44–4.02)

*SARS-CoV-2* severe acute respiratory syndrome coronavirus 2, *OR* odds ratios, *CI* confidence intervals.

The frequencies of AA, GA, and GG genotypes in *BsmI* rs1544410 polymorphism in the Alpha variant were 430 (42.1%), 507 (49.6%), and 85 (8.3%), respectively. These frequencies in the Delta variant was 426 (41.7%), 479 (46.7%), and 121 (11.6%), respectively. In the Omicron BA.5 variant, the frequency of CC was 420 (37.0%), CT was 646 (56.9%), and TT was 70 (6.1%) (Fig. 2B).

When we adjusted the effect of *BsmI* rs1544410 polymorphism for SARS-CoV-2 variants, the COVID-19 mortality rate was related to *BsmI* rs1544410 GA (OR 3.26, 95% CI 2.49–4.27) in the Alpha variant and with *BsmI* rs1544410 GG (OR 5.08, 95% CI 3.77–6.84*)* and GA (OR 3.26, 95% CI 2.09–5.10*)* in the Delta variant and with GG in the Omicron BA.5 variant (OR 2.41, 95% CI 1.44–4.02) (Table 3).

According to our findings, the C-A haplotype was observed to be the predominant form among all SARS-CoV-2 variants. The A-G haplotype was linked with COVID-19 mortality in both the Alpha (OR 1.56, 95% CI 1.27–1.92*)* and Delta (OR 2.70, 95% CI 2.15–3.38*)* variants. The A-A haplotype for the Omicron BA.5 (OR 10.37, 95%CI 4.40–24.46) variants was statistically significant (Table 4). There were strong LD between *ApaI* rs7975232 and *BsmI* rs1544410 (r2 = 0.91).

**Table 4:** SARS-CoV-2 variants and *ApaI* rs7975232 and *BsmI* rs1544410 haplotypes.

Haplotypes	Frequency	Alpha	Delta	Omicron
		OR (95% CI)	OR (95% CI)	OR (95% CI)
CA	0.5998	1.00	1.00	1.00
AG	0.3113	1.56 (1.27–1.92)	2.70 (2.15–3.38)	0.79 (0.62–1.00)
AA	0.0573	–	0.71 (0.51–1.00)	10.37 (4.40–24.46)
CG	0.0317	–	–	–

*SARS-CoV-2* severe acute respiratory syndrome coronavirus 2, *SNPs* single nucleotide polymorphisms, *OR* odds ratios, *CI* confidence intervals.

## Discussion

We investigated how the *ApaI* rs7975232 and *BsmI* rs1544410 affected the susceptibility to COVID-19 and showed that they might be used as genetic indicators for infection by different SARS-CoV-2 variants.

Alleles A (0.37) for the *ApaI* rs7975232 and G (0.34) for the *BsmI* rs1544410 polymorphisms as MAF were directly related to mortality in patients with COVID-19.

The MAF results in our study for *ApaI* rs7975232 were almost similar to different Asian populations, including Asian (0.313), East Asian (0.314), and other Asian (0.310), while it was different from South Asian (0.615), European (0.537), Latin American (0.589), and African (0.630) (https://www.ncbi.nlm.nih.gov/snp/rs7975232). The MAF for *ApaI* rs7975232 in deceased patients (0.42) was slightly higher than recovered patients (0.32) in our study.

The MAF result in our study for *BsmI* rs1544410 were similar to a study in Iran and South Asian (0.443), Latin American (0.366), European (0.398), and African (0.262), but was different from other regions including Asian (0.060), East Asian (0.056), and other Asian (0.076) (https://www.ncbi.nlm.nih.gov/snp/rs1544410). The MAF for *BsmI* rs1544410 in deceased patients (0.39) was slightly higher than recovered patients (0.30) in our study.

In this study, the levels of vitamin D in COVID-19 patients, especially those infected with the Delta variant with a higher mortality rate, were lower than the other two variants. It has been found that vitamin D can play an antiviral inhibitory role in nasal epithelial cells in SARS-CoV-2 infection^16^. This virus enters the host cells after binding to its receptors on the cell’s surface called ACE2 by Spike protein. Type II alveolar cells, in which ACE2 receptors are strongly expressed, are the virus’s primary target^17^. Calcitriol, a vitamin D agonist, increases the *ACE2* expression and soluble ACE2, which may lead to virus trapping and inactivation. The renin–angiotensin–aldosterone system, altered by SARS-CoV-2 infection, is negatively regulated by calcitriol, inhibiting renin expression. This increased availability of angiotensin II leads to tissue damage, inflammation, and multi-organ failure^18^.

Active forms of vitamin D and lumisterol have been shown to block SARS-CoV-2 replication machinery enzymes (main protease and RNA-dependent RNA polymerase), implying that novel vitamin D and lumisterol metabolites are potential antiviral therapeutic candidates. Moreover, these metabolites may prevent SARS-CoV-2 receptor binding domain from attaching to ACE2 by interacting with transmembrane serine protease 2 (TMPRSS2) and ACE2. The structural and dynamical motion alterations brought on by these interactions could impact TMPRSS2's ability to prime the SARS-CoV-2 spike proteins^19^. As a result, novel CYP11A1-derived vitamin D3 hydroxyderivative, including 20(OH) vitamin D3 and 20,23(OH)2 vitamin D3, and lumisterol hydroxymetabolites can inhibit COVID-19 via both independent and nuclear receptor-dependent mechanisms, making them excellent candidates for antiviral drug research as well as the informed use of their precursors as nutrients or supplements in the prevention and attenuation of COVID-19 disease^20,21^.

Vitamin D's active hydroxyl forms have anti-inflammatory and antioxidant effects, and they also boost innate defense to infectious agents. These characteristics are shared by non-calcemic hydroxyderivatives produced by CYP11A1 and calcitriol. They exhibit inverse agonism on the retinoic acid‐related orphan receptors-γ (ROR-γ), suppress the synthesis of pro-inflammatory cytokines, downregulate NF‐κΒ, and combat oxidative stress by activating transcription factor NF‐E2‐related factor 2 (NRF2). As a result, a direct delivery of vitamin D hydroxyderivatives deserves consideration in the therapy of COVID19 of various etiologies^22^.

Human VDR has more than 14 distinct identified polymorphisms. These polymorphisms may affect how VDR binds to calcitriol to modulate its response. *FokI* rs2228570, *BsmI* rs1544410, *ApaI* rs7975232, and *TaqI* rs731236 are the four SNPs that are most commonly examined. They were demonstrated independently modifying vitamin D status and in haplotypes^23^.

The COVID-19 death rate was considerably more significant in patients with the *ApaI* rs7975232 AA genotype than in other genotypes. The COVID-19 mortality rate was related to *ApaI* rs7975232 CA in the Alpha variant and with AA and CA in the Delta variant and with AA in the Omicron BA.5 variant. In agreement with our results, Apaydin et al. showed that the AA genotype was common among patients with severe COVID-19^24^.

Cohorts from Nigeria, Egypt, Ethiopia, Pakistan, Saudi Arabia, Lebanon, Turkey, and Italy were found to frequently have the AA genotype, according to the frequencies of the *ApaI* rs7975232 polymorphism. In contrast, the cohorts from Iran, the US, Poland, Greece, Mexico, India, the Netherlands, Czechia, Croatia, Russia, Spain, Finland, Brazil, and Tunisia frequently had the AC genotype. The CC genotype of the *ApaI* rs7975232 gene was most common in deceased patients from Korea, Japan, and China^25^. Studies with hepatitis B virus demonstrated that CA/AA genotypes of *ApaI* rs7975232 polymorphism trigger T helper 2 (Th2) cells proliferation, but there are no studies on *ApaI* rs7975232 and respiratory system viral infection. On the other hand, AA genotypes result in Th1 proliferation and anti-inflammatory cytokine production, which accelerates the progression of liver disease progression to cirrhosis^14^.

The fact that participants with the AA genotype of the *ApaI* rs7975232 polymorphism in this study had a greater death rate suggests that Th2 can also release Interleukin-6 (IL-6), which is related to COVID-19 prognosis. IL-6 is one of the key factors in the cytokine storm caused by COVID-19. IL-6 induces endothelial dysfunction with expression of tissue factor and adhesion molecules via upregulation of angiotensin converting enzyme-2 receptor. These negative effects of IL-6 were mitigated by vitamin D and VDR polymorphisms. As a result, it is possible that this is one of the putative mechanism(s) by which vitamin D exerts its positive effects in COVID-19 infection^26^. Subjects with the severe and moderate disease who had the "CA" genotype compared to "CC and AA" genotypes demonstrated a more severe risk, according to the study by Abdollahzadeh et al. Contrary to "CA and AA" and "CA" genotypes, symptomatic-asymptomatic and moderately-asymptomatic patients with the CC genotype were more likely to have signs and symptoms. In contrast to our findings, none of the deceased participants had the AA genotype^15^.

This study's patients with the GG genotype in this study exhibited a higher COVID-19 mortality rate in the *BsmI* rs1544410 polymorphism. The COVID-19 mortality rate was related to *BsmI* rs1544410 GA in the Alpha variant, *BsmI* rs1544410 AA and GA in the Delta variant, and GG in the Omicron BA.5 variant. It has been demonstrated that the *BsmI* rs1544410 G allele can be a risk factor for COVID-19 severity^15^, while no such relationship was seen in the study of Apaydin et al.^24^. The *BsmI* rs1544410 polymorphism's diversity revealed that the cohorts of the US, China, Poland, Turkey, Egypt, Italy, Saudi Arabia, Russia, Czechia, India, Greece, the Netherlands, Croatia, Brazil, Spain, Tunisia, Nigeria, and Lebanon frequently had the *BsmI* rs1544410 AG genotype. In contrast, the cohorts from Iran, Korea, Japan, Finland, Pakistan, and Mexico frequently had the *BsmI* rs1544410 GG genotype in deceased patients, but this was not significant^25^.

The association of *BsmI* rs1544410 and viral infections such as HIV has been investigated. It has been demonstrated that *BsmI* rs1544410 A-allele was strongly correlated with the rapid progression of HIV disease. It is unclear exactly how the *BsmI* rs1544410 G-allele confers protection, while the *BsmI* rs1544410 A-allele raises the likelihood of disease^27^. The *BsmI* rs1544410 G to A polymorphism alteration occurs in the 3' untranslated regions (3' UTRs) of the VDR gene and is hypothesized to affect the VDR messenger RNA stability. This polymorphism has been linked to an increased HIV infection susceptibility and faster rate of HIV disease development^28,29^.

Strong LD exists between *BsmI* rs1544410 and another 3′ UTR polymorphism (*ApaI* rs7975232), which has also been linked to the course of HIV illness. Given that the *BsmI* rs1544410 polymorphism is a synonymous mutation, the relationships seen may be explained by LD with one or more functional polymorphisms at other locations in the *VDR* gene^30^. However, synonymous rather than silent mutations could cause alternations in the protein's expression, conformation, and function. Therefore, *BsmI* rs1544410 polymorphisms might also directly change the VDR^31^. The findings that the *BsmI* rs1544410 A-allele is more influential in disease progression in the Delta variant than the other two may be explained by the difference in the serum vitamin D level, as these levels in patients with the Delta variant were much higher. Also, in this study, there was a strong LD between *BsmI* rs1544410 and *ApaI* rs7975232.

According to our findings, the C-A haplotype was more common among all SARS-CoV-2 variations. The A-G haplotype was linked with COVID-19 mortality in both the Alpha and Delta variants. The A-A haplotype for the Omicron variants was statistically significant. These two SNPs may likely function differently in distinct SARS-CoV-2 variants. However, the mechanism underlying this divergence remains unknown.

There were several limitations in our study that should be considered. We did not have any healthy controls who had not previously suffered from COVID-19. Besides, previous vaccination information of all patients was not available. Moreover, this study was conducted in only one population with the same ethnicity. To generalize the relationship between these two polymorphisms to the whole society, more studies should be done on different races in Iran.

In conclusion, our study showed that the serum vitamin D level and *BsmI* rs1544410 and *ApaI* rs7975232 polymorphisms were related to the mortality rate of SARS-CoV-2 with different variants. The COVID-19 mortality rate was related to *ApaI* rs7975232 CA genotype in the Alpha variant and with AA and CA genotypes in the Delta variant and with AA genotype in the Omicron BA.5 variant. Moreover, in *BsmI* rs1544410 polymorphisms, the mortality rate was correlated with GA genotype in the Alpha variant and with GG and GA genotypes in the Delta variant and with GG genotype in the Omicron BA.5 variant. The A-G haplotype was linked with COVID-19 mortality in both the Alpha and Delta variants. The A-A haplotype for the Omicron BA.5 variants was statistically significant. Further studies in different ethnicities should be done to confirm our results.

## Data Availability

All data that support all the experimental findings in this article is available in the paper.
